# The Biological Actions and Regulations of Lactic Acid-Linked Histone Lactylation

**DOI:** 10.3390/biology15100774

**Published:** 2026-05-13

**Authors:** Yanli Zhu, Kaiqi Li, Yiting Wang, Yueyao Li, Chuyang Zhu, Cuipeng Zhu, Long Yuan, Ping Hu, Haoyu Liu, Demin Cai

**Affiliations:** 1 College of Animal Science and Technology, Yangzhou University, Yangzhou 225009, China; liza.yl.zhu@outlook.com (Y.Z.); kqli77@outlook.com (K.L.); ytwang1631@outlook.com (Y.W.); yueyaolee2001@163.com (Y.L.); zhuchuyang@foxmail.com (C.Z.); cpzhu0318@outlook.com (C.Z.); yuanlong0715@outlook.com (L.Y.); pinghu@yzu.edu.cn (P.H.); haoyu.liu@yzu.edu.cn (H.L.); 2 International Joint Research Laboratory, Universities of Jiangsu Province of China for Domestic Animal Germplasm Resources and Genetic Improvement, Yangzhou 225009, China; 3 Guangling College, Yangzhou University, Yangzhou 225009, China

**Keywords:** lactic acid, histone lactylation, inflammation, tumor, neuropathy, gut microbe

## Abstract

Once written off as nothing more than a waste product of glycolysis, lactic acid is now seen as a key signaling molecule that operates across a wide range of physiological and pathological processes, from immune regulation and tumor metabolism to neural function. But its role goes beyond energy metabolism and cell signaling. Recent studies have uncovered a new type of post-translational modification called histone lactylation, in which lactate itself provides the lactoyl group attached to lysine residues on histones. This modification directly ties a cell’s metabolic state to the epigenetic control of gene expression. For example, histone lactylation helps shift macrophages from a pro-inflammatory M1 phenotype to an anti-inflammatory M2 phenotype by fine-tuning gene transcription. In this review, we walk through the discovery and biochemical foundation of histone lactylation; discuss the likely writer and eraser enzymes that manage its dynamic changes; and highlight recent advances in understanding the role of this modification in inflammation, tumorigenesis, neurological disorders, and interactions with gut microbes. We also lay out key unanswered questions and consider why targeting protein lactylation might open up new therapeutic possibilities.

## Simple Summary

Lactic acid was once thought to be nothing more than a waste product from energy production in the body. But new research shows that lactic acid can do something surprising. It can attach to histones, the proteins that help package and control DNA. This attachment, called histone lactylation, can switch specific genes on or off. In this review, we explain how this process works; what cellular mechanisms regulate it; and how it affects key body functions such as inflammation, tumor growth, nerve-related diseases, and the interaction between gut microbes and the body. Understanding histone lactylation is important because it links how cells produce energy to how genes are controlled. This knowledge may lead to new ways to treat diseases like cancer, sepsis, Alzheimer’s disease, and obesity-related conditions by targeting how cells use lactic acid.

## 1. Introduction

For a long time, lactic acid has been considered a waste product of anaerobic glycolysis. However, new data indicate that lactic acid is not only a key metabolite connecting glycolysis and oxidative phosphorylation but also a multifunctional biological signaling molecule [1]. On the one hand, lactate regulates both intracellular and extracellular metabolic processes throughout the body. On the other hand, it also has a variety of biological effects, including anti-inflammatory activity, immune regulation, and gene expression modulation [2].

Histones are central components of chromatin—a complex of DNA and proteins that organizes and regulates the genome. The C-terminal regions and N-terminal tails of histones undergo post-translational modifications, which play a crucial role in histone modification. Cell metabolism involves the uptake, release, and biochemical exchange of nutrients to generate energy and synthesize complex molecules. The intermediate and end products of metabolism also serve basic signaling functions, regulating cellular signaling and gene expression in response to nutrient availability. Core histones (H2A, H2B, H3, and H4) and linker histones (H1 and H5) are alkaline, positively charged proteins. One way that these metabolites emit signals is through chemical modifications of proteins such as histones. Zhang et al. described their discovery of a previously unknown histone modification, namely, histone lactylation, which uses lactate as the substrate [3]. The authors further demonstrated that lactic acid promotes epigenetic regulation of genes by adding lactoyl groups to histone lysine residues, and that lactate serves as the precursor for histone lysine lactylation (Kla), which stimulates gene transcription from chromatin [3].

Recent studies increasingly suggest that lactylation functions as a broader metabolic–epigenetic regulatory switch rather than simply a downstream consequence of lactate accumulation. Beyond histones, emerging evidence indicates that non-histone lactylation participates in the regulation of multiple biological processes, including metabolic enzyme activity, signal transduction, protein stability, transcriptional regulation, and immune responses [4,5]. In parallel, recent structural and mechanistic studies have begun to explore how lactylation marks are selectively recognized and interpreted by chromatin-associated factors and regulatory proteins, providing new insights into the molecular “decoding” mechanisms of lactylation signaling [6].

The biological functions of lactylation are highly context-dependent and may vary across different cell types, tissues, and disease stages. In certain pathological conditions, lactylation may promote tumor progression or immune suppression, whereas in other contexts, it may contribute to tissue repair and restoration of homeostasis [7,8]. This complexity highlights both the therapeutic potential and the translational challenges of targeting lactylation pathways. In addition, growing evidence suggests that the functions of lactylation are influenced by cell type, metabolic state, tissue microenvironment, and site-specific chromatin regulation [4,9].

Therefore, a comprehensive understanding of lactylation biology requires not only characterization of its molecular mechanisms but also clarification of its context-specific functions and regulatory networks. In this review, we summarize the current knowledge regarding the generation, regulation, and biological functions of lactylation, with particular emphasis on its roles in inflammation, cancer, fibrosis, stem cell biology, and metabolic diseases. We also discuss current technical limitations, emerging perspectives, and future challenges in lactylation research.

## 2. Sources and Removal of Lactic Acid

Lactate, as a hydroxy-carboxylic acid, exists as two isomers in mammals: L-lactate and D-lactate [10]. The source of D-lactic acid is primarily daily food, with a small portion produced by microbial fermentation of undigested carbohydrates in the stomach [11]. L-lactic acid is the main isomer under physiological conditions. In mammals, the main source of intracellular L-lactic acid is pyruvate from the conversion of glucose and alanine [12,13]. Glucose is the main source of energy and carbon for mammalian cells. It not only provides ATP but also generates various metabolites that serve as substrates for anabolic pathways [14]. In normal cells, glucose transporters transport glucose into the cell, where it is metabolized into pyruvate through catalysis by various glycolytic enzymes. Pyruvate enters the mitochondrial matrix and, under sufficient oxygen conditions, is converted into acetyl-CoA (CoA) by the pyruvate dehydrogenase (PDH) complex; acetyl-CoA then enters the tricarboxylic acid (TCA) cycle for oxidative phosphorylation (OXPHOS), producing large amounts of ATP available to the body. However, when intracellular oxygen is insufficient, pyruvate is converted by lactate dehydrogenase (LDH) into lactic acid and two ATPs [15]. In the 1920s, Otto Warburg discovered specific changes in the energy metabolism of tumor cells: even under aerobic conditions, glucose metabolism in tumor cells shifts toward glycolysis. That is, the rates of glucose uptake and glycolysis increase, producing more glycolytic intermediates and a large amount of ATP. This results in the phenomenon of “aerobic glycolysis,” now known as the “Warburg effect” [16,17]. The Warburg effect increases the rate of lactic acid production and acidifies the tumor microenvironment, thereby creating favorable conditions for the survival of cancer cells.

L-lactic acid is cleared by two pathways in the body. On the one hand, L-lactic acid is oxidized by LDH to pyruvate, and NAD^+^ is reduced to NADH. The pyruvate produced by lactic acid oxidation enters the mitochondria and is converted by the pyruvate dehydrogenase complex (PDC) into acetyl-CoA, generating one molecule of NADH. Acetyl-CoA enters the tricarboxylic acid (TCA) cycle and is utilized. Pyruvate, on the other hand, is converted by pyruvate carboxylase to oxaloacetic acid, which is then used within the body through the gluconeogenic pathway to synthesize endogenous glucose. Inborn or acquired impairments of these two lactic acid metabolic pathways in vivo lead to the obstruction of the production of ATP by mitochondrial aerobic respiration. As a result, cells rely on the glycolytic pathway, resulting in L-lactic acid accumulation [18,19,20,21,22,23].

## 3. Lactate Transporters and Receptors

The pKa of lactic acid is 3.86, indicating that it exists in the form of anions at physiological pH and cannot be transported inside and outside of the cell membrane by free diffusion. The intracellular transport of lactic acid is determined by the concentration gradient, pH gradient, and redox state. The monocarboxylic acid transporter (MCT) family is composed of 14 transmembrane proteins encoded by the SLC16A gene family [24]. Each subtype is distributed in different tissues and has tissue specificity. Four members of the family—MCT1, MCT2, MCT3, and MCT4—are responsible for the proton-coupled transport of several monocarboxylic acid metabolites, such as pyruvic acid, L-lactic acid, and ketone bodies (acetoacetic acid and D-β-hydroxybutyrate), across the plasma membrane [25]. MCT1 and MCT4 are mainly responsible for the transport of lactic acid. MCT1 is present in most tissues that produce lactic acid, and it primarily promotes the uptake and transport of lactic acid [26]. However, in glycolytic cancer cells and other specific tissues (such as white muscle fibers and astrocytes), MCT4 is superior to MCT1 in lactate transport [23,27,28,29]. In contrast, MCT2 has a higher affinity for lactate, but its tissue distribution is limited, being mainly distributed in tissues such as neurons and renal tubules, promoting efficient lactate uptake under low-concentration conditions [30]. Therefore, MCT1 and MCT4 are responsible for systemic lactate flux, while MCT2 plays a specialized role in high-affinity lactate transport in specific cell types. MCT3 has similar kinetic characteristics to MCT1, but the expression of MCT3 is limited to the basement membrane of retinal pigment epithelial cells and choroid plexus epithelial cells [31]. Its main function is to promote the transport of lactic acid produced by glycolysis [32]. The MCT family plays a vital role in maintaining many biological functions, such as cell metabolism, acid–base balance, and redox balance, and especially in regulating the rapid transmembrane transport of mono-carboxylic acids such as glycolytic metabolites, ketone bodies, and short-chain fatty acids [33,34].

G-protein-coupled receptor 81 (GPR81) belongs to a subfamily of GPCRs, known as hydroxy-carboxylic acid receptors (HCARs), which is highly homologous to GPR109a and GPR109b [35,36]. Notably, GPR81 is currently considered the primary and classical receptor mediating lactate signaling in metabolic tissues. As the only known endogenous lactate receptor under physiological conditions, it is distributed in adipose tissue, the kidney, the liver, skeletal muscle, the brain, the pancreas, and other tissues and organs, with its expression level in adipose tissue being particularly high [37,38]. GPR81 is activated at high blood lactate levels, which can enhance cerebrovascular endothelial growth factor A (VEGFA) and cerebrovascular production [39]. Cyclic adenosine monophosphate (cAMP), as a second messenger, is a key regulator that initiates adipocyte lipolysis when glucose is sufficient in normal tissues. Studies have found that lactic acid binds to GPR81 in an autocrine manner, resulting in decreased cAMP and inhibition of lipolysis [40]. GPR81 expression is significantly reduced in ob/ob mice, an animal model of type 2 diabetes characterized by high inflammation. Under normal physiological conditions, lactate inhibits the cAMP signaling pathway through GPR81, limiting the release of free fatty acids (FFAs) and thus inhibiting the lipolysis process. However, in pathological states such as obesity and type 2 diabetes, chronic low-grade inflammation inhibits the expression of GPR81, and the decrease in GPR81 expression weakens the signal mediated by lactic acid, thus relieving its inhibition on lipolysis, enhancing lipolysis, increasing FFA release, and intensifying insulin resistance and systemic inflammation. Therefore, despite the high level of lactic acid, the downregulation of GPR81 in obesity/diabetes promotes lipolysis and aggravates the deterioration of metabolism rather than playing a protective role [41].

## 4. The Function of Lactic Acid

### 4.1. Lactic Acid Provides Energy

Lactic acid, once considered a product of anaerobic glycolysis, has long been regarded as a metabolic waste product harmful to the body; however, it can also serve as an energy substrate to provide energy for the body [42,43]. The astrocyte–neuron lactate shuttle (ANLS) hypothesis proposed by Pellerin and Magistretti supports that lactic acid produced by the astrocyte glycolysis pathway is preferentially utilized by neurons to provide energy for excitatory neurotransmission [23,44]. In the brain, lactic acid can serve as an alternative fuel for synaptic signaling when glucose is scarce. When hypoxia or ischemia occurs in the brain, the neurotransmitter glutamate induces an increase in the glycolytic activity of astrocytes, leading to the secretion of substantial amounts of lactic acid into the synaptic clefts, where neurons can use it as a substitute for or complement to glucose to maintain brain function. In addition, Avital Schurr et al., using electrophysiological and biochemical methods to prepare rat hippocampal sections, demonstrated that lactic acid provides energy support when neurons are activated and protects the nerves by resisting oxidative stress [45]. Glucose is generally considered the primary energy substrate for the brain under physiological conditions [46]. However, increasing evidence suggests that lactate can also serve as an important alternative energy source. Under resting conditions, the contribution of lactate to total brain energy metabolism is relatively limited. In contrast, during periods of intense neuronal activity, exercise, or metabolic stress, lactate utilization is significantly enhanced, and it may account for approximately 20–30% or more of oxidative metabolism in certain brain regions [47,48].

The extent to which lactate contributes to brain energy metabolism remains a subject of ongoing debate. This controversy is partly driven by differing experimental models and interpretations of the astrocyte–neuron lactate shuttle (ANLS) hypothesis [49], which proposes that astrocyte-derived lactate is transferred to neurons as a preferred fuel. While some studies support a substantial role for lactate, others emphasize the dominant role of glucose, highlighting the context-dependent nature of brain energy metabolism [46].

At present, the consensus is that, while glucose remains the obligatory primary energy substrate for the brain under physiological conditions, lactate serves as an important supplemental and context-dependent fuel, particularly during periods of high energy demand, metabolic stress, or glucose scarcity. The relative contribution of lactate to brain energetics appears to increase in proportion to circulating lactate levels, but the extent to which endogenous (astrocyte-derived) lactate supports neuronal energy demands under normal physiological conditions continues to be an active area of investigation.

### 4.2. Lactic Acid Acts as a Signaling Molecule

Lactic acid acts as an important signaling molecule in the central nervous system and has been implicated in the regulation of sleep, circadian rhythms, and mood-related behaviors, including antidepressant-like effects [50]. Experimental studies have shown that intraperitoneal administration of high doses of lactate in mice is associated with reduced locomotor activity and increased peripheral blood lactate levels [50]. One possible explanation for the observed elevation in blood lactate levels is the accumulation of lactate resulting from prolonged muscle activity under conditions of insufficient oxygen supply, which represents a well-established metabolic response [43]. Therefore, it is speculated that a possible explanation for the decrease in activity may be that systemic lactate injection causes muscle pain.

Transforming growth factor β2 (TGFβ2) expression can be induced by transforming growth factor β2 (TGFβ2) to produce a large amount of lactic acid in tumor cells through the Warburg effect. This enhances the activation of matrix metallopeptidase-2 (MMP-2) and increases the metastasis of glioma [51].

Lactic acid plays an important role as a signaling molecule in adipose tissue macrophage (ATM) polarization and the development of obesity-related insulin resistance [52]. Exercise-induced lactic acid promoted the secretion of adipokine converting growth factor β2 (TGF-β2), thereby alleviating the elevated glucose levels in the blood of mice fed a high-fat diet (HFD). Therefore, a moderate increase in plasma lactate concentration may have a positive effect on the health of organisms [51].

In addition to GPR81, lactate can also act through GPR132, a receptor whose role is context-dependent rather than related to systemic metabolic regulation; it is mainly involved in immune regulation, especially in regulating macrophage polarization through the cAMP PKA signaling pathway [53]. Overall, there is a division of labor between tissue specificity and functional specificity. GPR81 mainly mediates the metabolic regulation of lactate, while GPR132 participates in its immune regulatory function in specific cellular environments. Administration of lactic acid at a dose of 800 mg/kg/day inhibits ATM proinflammatory M1 polarization by activating the GPR132-PKA-AMPKα1 signaling pathway, thereby improving insulin resistance in HFD-fed mice. These studies suggest that lactic acid plays a key role in alleviating obesity-related type 2 diabetes [52].

In the tumor microenvironment, lactic acid produced by tumor cells is phagocytosed by macrophages and, mediated by HIF-1α, affects the proliferation of tumor cells, induces VEGF expression, promotes the expression of the M2 macrophage polarization marker gene arginase I (Arg1), and enables tumor cells to continue to grow [54]. Another study also found that lactic acid is the factor driving the transformation of macrophages into an anti-inflammatory phenotype in the tumor microenvironment, where it can mediate immunosuppressive effects [55].

## 5. Histone Lactylation: Discovery, Biochemical Basis, and Chromatin Regulation

### 5.1. Discovery and Biochemical Basis

Epigenetic mechanisms include DNA methylation, histone modification, and microRNA (miRNA), which can produce heritable phenotypic changes without changing the DNA sequence [56]. Histone modification is one of the important mechanisms of epigenetic modification. The histone complex consists of a tetramer composed of proteins H2A, H2B and H3 and H4 in chromatin, which is wrapped by 147 bp of DNA to form a nucleosome [56]. Their various post-translational modifications (PTMs) regulate gene expression. Some classical post-translational modifications of histones include acetylation, methylation, ubiquitination, phosphorylation, and succinylation. These PTMs regulate many processes in cell physiology and biochemistry by disrupting the spatial structure of proteins. In 2019, Zhang et al. [3] identified a novel post-translational histone modification termed lactylation (Kla). Zhang et al. first found a mass shift of 72.021 Da on lysine residues in human cancer cells using mass spectrometry, which was speculated to result from a lactoyl group added to the lysine residues [3]. Subsequently, isotope metabolism labeling techniques and a variety of in vitro and in vivo experiments were used to verify the widespread existence of lactylation modification of histone lysine residues. In many known histone lysine modifications, the substrate is derived from the corresponding metabolic substances, such as acetyl-CoA for acetylation and succinyl-CoA for succinylation. The histone lactylation discovered by Zhang et al. is derived from lactic acid, and intracellular lactate production affects the level of histone lactylation in a dose-dependent manner.

In addition to the active enzymatic reaction that transfers lactoyl groups from lactoyl CoA to lysine residues, Gaffney [56] and Varner et al. [57] also demonstrated by mass spectrometry that proteins can obtain lysine lactylation from lactoyl glutathione (LGSH) via a non-enzymatic reaction. Methylglyoxal is a glycolytic byproduct that binds glutathione to form LGSH through the enzyme glyoxalase I (GLO1). GLO2 hydrolyzes LGSH to glutathione and D-lactic acid, thereby regulating LGSH levels. The study of Zhang et al. [3] focused on lactate modification of histones and clarified that it can be used as a novel epigenetic regulator of gene transcription. However, another study found that many metabolic enzymes are also modified by lactate, and lactate modification of related enzymes can achieve negative feedback regulation of the glycolysis pathway [58]. According to subsequent studies, lactate modification occurs on both histones and many non-histone proteins. Although there are still some unresolved disagreements, the discovery of protein lactylation not only opens up new areas for the study of post-translational modification of proteins but also suggests a potential molecular mechanism for the role of lactic acid in physiological and pathological processes such as tumorigenesis, metabolism, and immunity.

### 5.2. Multi-Level Regulation of Chromatin State by Histone Lactylation

Beyond its role in gene transcription, histone lactylation (Kla) actively remodels the chromatin structure across multiple levels of organization. Mechanistically, lactylation neutralizes the positive charge of lysine residues on histones, thereby reducing electrostatic interactions with the negatively charged DNA backbone and promoting a more open and accessible chromatin conformation [3]. This charge neutralization effect has been validated by studies showing that Kla enhances chromatin accessibility and facilitates transcription factor binding.

At the three-dimensional genome architecture level, histone lactylation, particularly H3K9la and H3K18la, has been associated with extensive chromatin remodeling. These changes include A/B compartment switching, topological-associated domain fusion, and the establishment of novel enhancer–promoter interactions [59,60]. Such structural alterations have been observed in metabolic disorders and are linked to the activation of specific gene programs, suggesting that lactylation can propagate metabolic signals into stable higher-order chromatin conformation changes.

Genome-wide profiling has revealed that H3K18la marks active CpG island-containing promoters of highly expressed genes and, more notably, tissue-specific active enhancers [61]. This distribution pattern resembles that of H3K27ac, a canonical active chromatin mark. While H3K18la does not define an entirely novel chromatin state distinct from known categories, its unique dependency on lactate links the cellular metabolic state directly to epigenetic marking. This positions H3K18la as a distinctive “metabolic–epigenetic sensor” that operates within the framework of active chromatin.

A separate line of inquiry has uncovered a phase separation-dependent mechanism for sustainable histone lactylation. In pathological angiogenesis, the intrinsically disordered region of semaphorin 6A forms liquid–liquid phase separation condensates that recruit RHOA and p300, facilitating p300 phosphorylation and driving a self-sustaining lactylation cycle [62]. This finding reveals that lactylation can be maintained through biomolecular condensates, adding another layer of regulatory complexity.

Regarding the distribution of lactylation along gene bodies, current evidence remains limited. All available datasets have mapped lactylation primarily to promoter and enhancer regions, with limited characterization of distribution within gene bodies [3,61]. No study has yet reported the presence of single or prolonged stretches of lactylated exons. It is important to clarify that lysine lactylation modifies histone proteins rather than DNA or RNA sequences; thus, the concept of “lactylated exons” refers to chromatin regions rich in Kla that coincide with exonic sequences, not direct modification of the exons themselves. Future high-resolution mapping, possibly combined with long-read sequencing technologies, will be required to determine whether Kla exhibits exon-specific enrichment or alternative splicing-associated distribution patterns.

It should be emphasized that histone lactylation is not restricted to a few lysine residues. Rather, it is a pan-histone modification observed on all four core histones and on linker histone H1. The specific sites vary with cell type, metabolic state, and pathological condition. Although H3K18la and H3K23la are the most frequently cited examples, histone lactylation occurs on multiple lysine residues across all core histones (H2A, H2B, H3, and H4) and even on linker histone H1. Table 1 summarizes the most consistently reported sites, but ongoing research continues to expand this list.

## 6. Loading and Clearing Mechanism of Histone Lactylation

### 6.1. Enzymatic Regulation of Histone Lactylation

**Figure 1:**
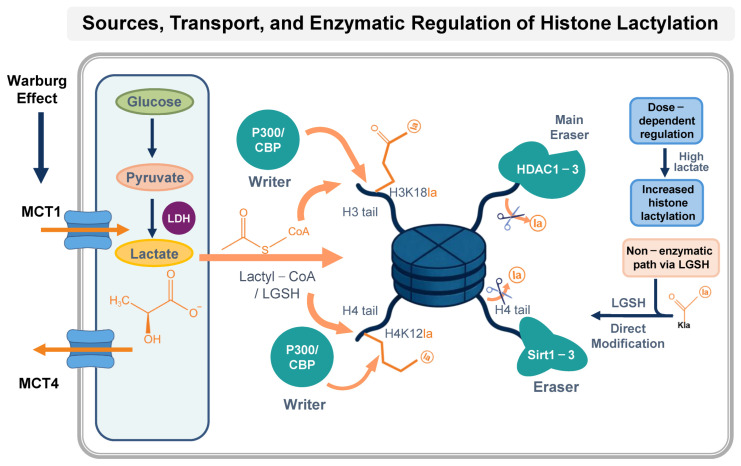
Sources, transport, and enzymatic regulation of histone lactylation. Glucose is converted to pyruvate via glycolysis, followed by lactate production catalyzed by lactate dehydrogenase (LDH). Lactate enters cells via monocarboxylate transporter 1 (MCT1). Lactate can be converted to lactyl-CoA or directly modify histones through p300/CBP-dependent or LGSH-mediated non-enzymatic pathways, leading to lysine lactylation (Kla). Delactylation is mainly mediated by HDAC1-3 and Sirt1-3. H3K18la is a representative modification site.

Lysine acylation is characterized by widespread occurrence and high evolutionary conservation in cells, and its changes are often reversible [69]. Since lysine lactylation modification is detected after histone H4 and LGSH are co-incubated, LGSH provides lactoyl groups for lysine, and this process does not require enzyme participation [56]. To summarize the sources of lactate, its transport, and the enzymatic regulation of histone lactylation, we present a schematic overview (Figure 1). Unlike histone lactylation, which does not require enzymes, most of the acylation of lysine residues requires specific proteins for “loading” and “clearing”. Histone acetylation and histone lactylation have many similarities. Studies have found that histone acetyltransferase (HAT) and histone deacetylase (HDAC) play a role in the dynamic regulation of histone lactylation. After overexpression of the coding gene EP300 of histone acetyltransferase p300 in human embryonic kidney HEK293T cells, it was found that the level of histone lactylation was increased, while knockdown of p300 in human colon cancer HCT116 cells and HEK293T cells reduced the level of pan-lactylation modification of histones and H3K18la [56]; in macrophages derived from mouse bone marrow, the same phenomenon can be observed when p300 is knocked down [70].

Research findings of Yang et al. [71] show that lactic acid induced HMGB1 and thereby increased the level of intracellular lactate. This phenomenon can be significantly antagonized by the inhibitor C646 of the p300/cyclic adenosine monophosphate response element binding protein (CBP). Knocking down p300 or CBP can also significantly inhibit this phenomenon. In addition, in vitro experiments on recombinant chromatin histone modification and transcription found that histone H3 and H4 lactylation levels were co-promoted by p53 and p300 [72]. Although it is not possible to prove the indirect effect of p300 and CBP in cells or that their enzymatic effect in vitro increases the level of histone lactylation, the above results show that p300 and CBP have the potential to serve as histone lactylation loading proteins.

HDACs rely on Zn^2+^ for their function and have three types: type I (human HDAC1–3 and HDAC8), type II (human HDAC4–7 and HDAC9–10), and type IV (human HDAC11). In contrast, class III HDACs, also known as sirtuins (human SirT1–7), rely on NAD as a co-substrate for the substitution reaction [73]. The study found that HDAC1-3 and Sirt1-3 are the most effective lysine lactylation-modified cleaning proteins in vitro. Overexpression and knockdown experiments also showed that HDAC1 and HDAC3 are the main deacetylase enzymes in cells. Sirt1-3 has a weak effect as deacetylase in in vitro experiments.

### 6.2. Key Technical Challenges in Lactylation Research

Despite the rapidly expanding interest in protein lactylation, the field faces several technical challenges that can complicate data interpretation and limit reproducibility. A clear understanding of these limitations is essential for designing rigorous experiments and for critically evaluating published findings.

The overall abundance of lactylation is relatively low. It has been documented that lactylation typically occurs at low stoichiometry, and its signal can be easily confounded with other modifications during mass spectrometric interpretation [1]. Furthermore, lactate can induce both histone and non-histone lactylation, contributing to various pathological processes [1]. This low abundance inevitably requires the application of high-resolution mass spectrometry coupled with effective enrichment strategies for accurate detection and reliable quantitative characterization [74,75].

Non-enzymatic mechanisms also contribute substantially to the lactylation landscape. The reactive glycolytic byproduct methylglyoxal is rapidly conjugated to glutathione via glyoxalase 1, generating lactoylglutathione. It has been shown that lactoylglutathione can directly transfer its lactate moiety to protein lysine residues, producing a lactoyllysine modification on proteins independently of dedicated lactyltransferases [56]. This non-enzymatic pathway can also be regulated by sirtuin 2 [76] and is enriched on glycolytic enzymes, thereby influencing glycolytic flux. In cells lacking glyoxalase 2, elevated lactoylglutathione levels lead to a marked increase in this modification, challenging the interpretation of lactylation signals derived solely from lactate supplementation [76].

Another layer of complexity arises from lactate chirality. Lactate exists as two enantiomers, L-lactate and D-lactate. L-lactate is the predominant form under physiological conditions, but D-lactate can also be generated from methylglyoxal via the glyoxalase pathway. Conventional lactylation detection workflows have traditionally not discriminated between these enantiomers. A recent study introduced a chemical derivatization method combined with HPLC-MS/MS to successfully separate L-lactyl-lysine, D-lactyl-lysine, and N-ε-(carboxyethyl)-lysine. This work demonstrated that L-lactyl-lysine, rather than D-lactyl-lysine or N-ε-(carboxyethyl)-lysine, is the primary lactylation isomer on histones and is dynamically regulated by glycolysis, whereas the other two forms become observable only when the glyoxalase system is compromised [74].

Antibody-based detection, while widely used, also has limitations. The specificity of lactylation antibodies requires careful validation because they can potentially cross-react with other acyl modifications. The commercial monoclonal antibody recognizing L-lactyl-histone H3 at Lys23 has been reported to exhibit minor cross-reactivity with L-lactyl-histone H3 at Lys18 (PTM Bio product specification). Therefore, orthogonal validation methods such as high-resolution mass spectrometry and peptide competition assays should be routinely employed to confirm antibody-based findings. Pan-lactylation antibodies may additionally detect D-lactyl modifications and may cross-react with other short-chain acylations.

A further technical hurdle is the isobaric mass overlap with other acylations. Lactylation has the same nominal mass increase of 72.021 Da as other acyl modifications, including β-hydroxybutyrylation, which has been identified as a novel histone mark in health and disease [77,78]. Without high mass accuracy, typically set to 3 parts per million or better, and without appropriate chromatographic separation, a peptide bearing lactylation cannot be reliably distinguished from one bearing these other acyl modifications. High-resolution mass spectrometry provides the necessary mass accuracy, but false positives remain a concern unless complemented by orthogonal strategies such as synthetic peptide standards and retention time alignment.

The confident assignment of lactylation to a specific lysine residue also requires rigorous quality control. When multiple lysine residues co-exist within the same peptide, MS/MS spectra may localize the modification to an incorrect residue. A false discovery rate threshold of 1 percent should be applied at both the peptide and site levels. High-confidence lactylation sites should be selected based on peptide scores and site localization metrics such as PTM scores [75]. Moreover, the presence of a characteristic cyclic immonium ion of lactyl-lysine can be used as an additional filter to improve assignment confidence. In some commercial analytical workflows, recommended mass tolerances for precursor and fragment ions are 10 parts per million and 0.02 Daltons, respectively, to ensure data quality across various sample types and buffers [74].

Taken together, these technical challenges highlight the necessity of integrating multiple complementary approaches, including high-resolution mass spectrometry with appropriate quality control, stereoisomer-resolving workflows, orthogonal validation strategies, and rigorous experimental controls. Such integration will help ensure that lactylation research advances on a robust and reproducible foundation.

## 7. The Role of Histone Lactylation in Different Physiological and Pathological Processes

### 7.1. Histone Lactylation Regulates Cell Fate

Cell fate is a complex and precise process coordinated by a variety of regulatory modes such as metabolomics, transcriptomics, and epigenetics [79,80,81]. The maternal transcription factor GLIS family zinc finger 1 (Glis1) is expressed only in eggs and fertilized eggs and is a powerful determinant of cell fate. Li et al. demonstrated a novel “epigenome–metabolome–epigenome” signaling cascade, in which the transcription factor Glis1 regulates pluripotent stem cell fate through coordinated metabolic and epigenetic reprogramming [82]. Specifically, Glis1-induced metabolic remodeling, including increased lactate production, feeds back to modulate epigenetic modifications and thereby promotes the acquisition of pluripotency. This mechanism not only enhances the reprogramming efficiency of somatic cells but also facilitates the reprogramming of aged cells and contributes to improved genomic stability in induced pluripotent stem cells (iPSCs) [82,83]. The results showed that Glis1 is a powerful determinant of cell fate. Glis1 could turn off the expression of somatic genes and turn on the expression of glycolytic genes. Glis1 promotes the metabolic remodeling of cells from mitochondrial oxidative phosphorylation to glycolysis, resulting in the production of substantial amounts of lactic acid and the promotion of histone lactylation (H3K18la) and the expression of pluripotent genes, thus improving the reprogramming rate and even realizing the reprogramming of aging cells [82]. Lactic acid was added to mouse embryonic stem cells cultured in vitro, and it was found that lactic acid could promote the modification of H3K18la in the promoter region of mouse cleavage embryo genes and embryo-related genes, as well as the transcription of these genes [84]. These studies have clarified the key role of histone lactylation modification in the process of cell fate determination.

The dynamic change of histone post-translational modification is an important background for epigenetic reprogramming of preimplantation embryos after fertilization [85]. Yang et al. detected histone pan-lactylation, H3K18la, and H3K23la in mouse oocytes, fertilized eggs, and preimplantation embryos. Histone lactylation in preimplantation embryos is related to the oxygen concentration in the culture environment. Hypoxia during in vitro culture will reduce histone lactylation and damage the development potential of preimplantation embryos [86]. Histone lactylation not only affects embryo development but also participates in the regulation of endometrial receptivity to embryos [87]. The level of H3K18la in the endometrium of sheep with successful pregnancy increased significantly, whereas that in sheep with failed pregnancy decreased significantly. This result suggests that enhanced glycolysis plays a significant role in stimulating endometrial remodeling. The lactic acid produced by glycolysis during embryo implantation can promote histone lactylation in the endometrium through the maternal–fetal interface, and it can apparently regulate the expression of endometrial genes related to endometrial redox homeostasis and cell apoptosis, thus reshaping the receptivity of the endometrium to embryos and promoting their successful implantation [86]. This finding provides new insights for developing potential clinical intervention strategies to improve the pregnancy rate after natural and assisted pregnancy.

### 7.2. Histone Lactylation and Inflammation

**Figure 2:**
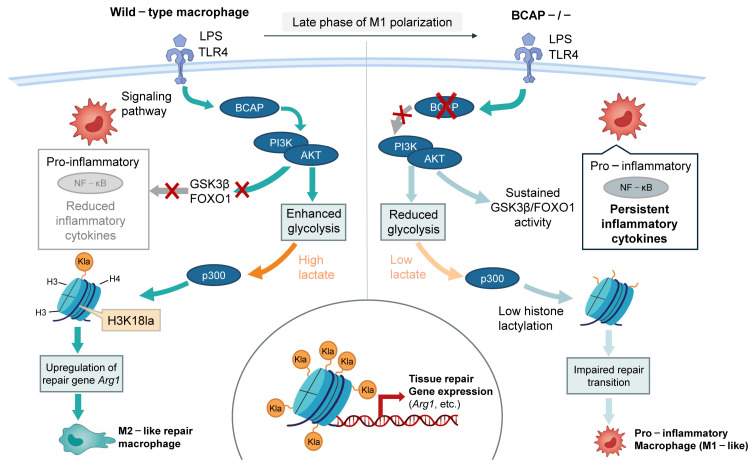
Histone lactylation drives macrophage transition from inflammation to tissue repair. In wild-type macrophages, BCAP-mediated PI3K-AKT signaling promotes glycolysis and lactate production, leading to p300-dependent H3K18la upregulation of repair genes (e.g., *Arg1*), driving the M2-like repair phenotype. In BCAP-deficient macrophages, glycolysis is impaired, lactate levels drop, histone lactylation is reduced, and repair transition is blocked, resulting in sustained inflammation.

Inflammation is the basis of various physiological and pathological processes [87]. Macrophages prevent inflammation by remodeling tissue and removing cell debris. Activated macrophages are usually divided into the M1 type, which participates in the pro-inflammatory response, or the M2 type, which participates in the anti-inflammatory response. The glycolytic pathway of M1-type macrophages is often highly active [88]. BCAP functions as an important adaptor in TLR signaling by activating the PI3K-AKT pathway. Upon TLR stimulation, BCAP-mediated PI3K-AKT signaling promotes glycolysis and lactate production in macrophages. The increased lactate subsequently enhances histone lactylation, particularly H3K18la, which drives the transcription of tissue repair-related genes such as Arg1. This process facilitates the transition of macrophages from a pro-inflammatory (M1) phenotype to a reparative (M2-like) phenotype [3]. In contrast, deficiency of BCAP impairs PI3K-AKT signaling, leading to reduced glycolysis and lactate production. Consequently, histone lactylation levels decrease, resulting in diminished expression of repair-associated genes and a failure to transition toward the reparative phenotype, thereby sustaining inflammation (Figure 2) [89].

Stimulation of PRRs, such as Toll-like receptors (TLRs), leads to macrophage activation and pro-inflammatory production. PI3K B-cell adapter (BCAP) is a new TLR signal adapter, which mediates the activation of the PI3K-AKT pathway after TLR connection. BCAP conducts TLR signals to the PI3K-AKT-GSK3β-FOXO1 pathway, which is associated with negatively regulating inflammation, but this function is not related to its role in promoting macrophage repair and transformation. This metabolite promotes histone lactylation, which is important for upregulation of repair genes and overall repair transformation of macrophages [8]. When the body loses BCAP, it leads to an excessive inflammatory reaction after TLR activation. Chemical inhibition or genetic elimination of PI3K or AKT has been shown to enhance the secretion of inflammatory cytokines [90,91,92,93]. As GSK3β and FOXO1 enhance NF-κB nuclear function, their phosphorylation and inhibition of AKT lead to decreased transcription of NF-κB-dependent inflammatory cytokines [90,91]. Macrophages show impaired lactate production, reduced histone lactylation, and reduced expression of tissue repair genes, which leads to a slow repair transition [8]. Figure 2 illustrates how the BCAP-PI3K-AKT signaling axis links lactate production to histone lactylation and drives the transition of macrophages from an inflammatory to a tissue-repair phenotype.

An important characteristic of pulmonary fibrosis is enhanced glycolysis, leading to an increase in the production of lactate, a byproduct of glycolysis. In this context, lactate can induce histone lactylation in alveolar macrophages and upregulate the expression of genes such as Arg1, thereby exacerbating the fibrosis process rather than simply inhibiting inflammation [70]. It should be pointed out that, although the polarization of M2-like macrophages is usually associated with anti-inflammatory responses and helps to reduce inflammation in acute inflammation conditions, in chronic pathological states such as pulmonary fibrosis, these macrophages often exhibit profibrotic effects, promoting disease progression by promoting extracellular matrix deposition and tissue remodeling. Therefore, lactate-driven histone acetylation is more likely to promote fibrosis progression during this process rather than just inflammation resolution.

However, Yang et al. found that HMGB1 lactylation has an adverse effect on sepsis in a mouse model of multi-bacterial sepsis. Extracellular lactic acid enters the cell via the transport of MCTs and induces HMGB1 lactylation through a P300/CBP-dependent mechanism. Subsequently, lactic acid induces the accumulation of HMGB1 lactylation in the cytoplasm, promotes its localization in the lysosome, and releases it into endothelial cells via exocrine bodies; it then destroys endothelial adhesion and tight junction proteins, increases the expression of adhesion molecules, and increases endothelial permeability, resulting in endothelial barrier dysfunction [71]. In addition, the level of H3K18la in peripheral blood mononuclear cells of septic shock patients and the level of histone lactylation in tumor-related macrophages were also significantly positively correlated with the transcription level of Arg1 in corresponding cells [94]. This suggests that H3K18la may have a significant impact on the balance of septic inflammation. According to other studies, lactate is the result of macrophage activation rather than the cause. Arg1 induction by lipopolysaccharide depends on autocrine or paracrine interleukin-6 (IL-6), the IL-6 receptor, and signal transducer and activator of transcription 3 (STAT3) [95]. In conclusion, histone lactylation certainly plays a role in the development of inflammation, suggesting a new approach for treating inflammation.

Nevertheless, the role of lactylation in inflammation is not uniformly protective. In the context of polymicrobial sepsis, excessive lactate-induced HMGB1 lactylation in macrophages has been reported to promote exosomal release of HMGB1, thereby impairing endothelial barrier integrity and exacerbating systemic inflammation [71]. In contrast, under sterile inflammatory conditions such as pulmonary fibrosis, lactylation-mediated macrophage reprogramming may contribute to tissue repair and fibrosis resolution [3,70]. Thus, the net effect of lactylation critically depends on the pathological context, including cell type, lactate concentration, tissue microenvironment, and disease stage.

### 7.3. Histone Lactylation and Tumor

The growth environment is especially important for the development and growth of tumors, and it is affected by numerous factors including the metabolite lactic acid. In the past, lactic acid was considered a biomarker of malignant tumors. The most typical feature of the Warburg effect is that tumor cells produce a large amount of lactic acid [96]. It is plausible that the level of protein lactate in tumors may also be significantly increased. Recent studies have shown that histone lactylation modification is closely related to tumor cell proliferation [97]. Yu et al. found that the m6A reader protein YTH N6-methyladenosine RNA-binding protein 2 (YTHDF2), as a new oncogene in ocular melanoma, can be directly regulated by H3K18la. YTHDF2 contributes to tumorigenesis by degrading circadian rhythm regulator 1 (PER1) and TP53. This discovery provides a new histone lactylation target for the treatment of ocular melanoma [97].

Hepatocellular carcinoma (HCC) is one of the most common cancers in the world. Liver cancer stem cells (LCSCs) are directly involved in the progression and recurrence of HCC [98]. These cells promote the growth of primary cancer cells and secondary tumor metastasis after transplantation, leading to disease recurrence, and are therefore important diagnostic markers of hepatocellular carcinoma. A study found that, in LCSCs, the expression level of pan-Kla was positively correlated with malignant tumor markers CD133, BCL2, and LDHA, and the expression levels of H3K9la and H3K56la were correlated with tumor growth. The upregulation of histone lactylation promoted the carcinogenesis of HCC. Compared with HCC cells, histone lactylation in LCSCs increased, and the increase in histone lactylation effectively promoted the progression of HCC [99]. This study provides a new direction for the treatment of HCC.

Metabolic reprogramming is one of the markers of cancer cells [96]. In non-small-cell lung cancer cells, the mRNA levels of glycolytic enzymes (HK-1 and PKM) and TCA cycle enzymes (SDHA and IDH3G) were downregulated and upregulated by lactic acid, and the histone lactylation levels of the HK-1 and IDH3G promoters increased, indicating that lactic acid at least partially mediates gene expression in non-small-cell lung cancer through histone lactylation, thereby regulating cell metabolism [100].

Von Hippel–Lindau (VHL) mutation plays a key role in the pathogenesis of renal clear cell carcinoma (ccRCC) and is present in up to 90% of patients with ccRCC. A high level of lactic acid is the main marker of ccRCC, and there is a close relationship between VHL and its targeted hypoxia-inducible factor (HIF)-related pathway, as well as glycolysis-derived lactic acid. In patients with ccRCC, inactive VHL was positively correlated with histone lactylation, and the high level of histone lactylation suggested poor prognosis. Inactive VHL-triggered histone lactylation activates platelet-derived growth factor receptor β (PDGFRβ), thereby promoting the progression of ccRCC. In turn, PDGFRβ signal transduction has been shown to stimulate histone lactylation, thus forming a carcinogenic positive feedback loop in ccRCC. Inhibition of histone lactylation in vivo also leads to inhibition of ccRCC growth; when protein lactylation and PDGFRβ are inhibited at the same time, the proliferation efficiency of ccRCC cells is lower. This positive feedback loop between histone lactylation and PDGFRβ signal transduction may provide new therapeutic hope for patients with ccRCC [59].

**Figure 3:**
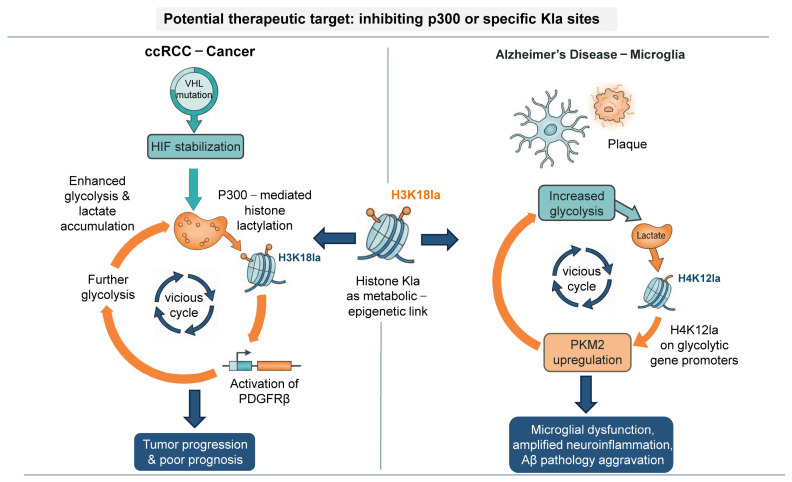
Histone lactylation forms pathogenic positive feedback loops in cancer and neurodegeneration. In clear cell renal cell carcinoma (ccRCC), VHL inactivation stabilizes HIF, enhancing glycolysis and lactate production, which promotes p300mediated H3K18la and activates PDGFRβ signaling, further driving tumor progression. In Alzheimer’s disease (AD), enhanced glycolysis in microglia leads to lactate-induced H4K12la, upregulating PKM2 and further boosting glycolysis, forming a metabolic–epigenetic positive feedback loop that exacerbates neuroinflammation and Aβ pathology.

However, the specific conditions and reasons for why histone lactylation modifications promote or inhibits gene transcription are not clear. Similar pathogenic positive feedback loops involving histone lactylation have also been identified in neurodegenerative diseases, as depicted in Figure 3.

It is worth noting that the biological effects of lactylation are highly context-dependent rather than uniformly beneficial or detrimental. In the tumor microenvironment, lactylation has been associated with immune suppression, tumor-associated macrophage polarization, and enhanced tumor progression [3,7]. However, the functional consequences of lactylation may vary substantially depending on tumor type, metabolic state, chromatin accessibility, and disease stage.

In contrast, under pathological conditions such as tissue injury and inflammation resolution, lactylation may contribute to tissue repair and restoration of tissue homeostasis [8]. In addition, the same lactylation mark, such as H3K18la, may promote oncogene expression in one cellular context while activating tumor suppressor-associated pathways in another, reflecting differences in chromatin accessibility and transcriptional cofactor recruitment. Therefore, the functional consequences of lactylation should be interpreted within specific biological contexts rather than as inherently protective or pathogenic. Global inhibition of lactylation may be beneficial in certain tumors but potentially detrimental in others. Further studies are required to clarify the cell type-specific and site-specific functions of lactylation before clinical translation.

### 7.4. Histone Lactylation and Neuropathy

Substantial evidence indicates that astrocyte-derived lactic acid can provide fuel for neurons and act as a signaling molecule by activating lactate receptors on the neuronal surface and modulating their function. Recent studies have found that lactylation, similar to lactic acid, also participates in nerve activity. Hagihara et al. [68] found that neuronal excitation and social frustration stress could upregulate the lactic acid level and protein lactylation modification level of brain cells. They identified 12 protein lactate modifications related to social frustration stress, including histone H1, and they also found that lower social behavior was related to higher histone H1 Kla. Alzheimer’s disease (AD) is the most common neurodegenerative disease. Microglia are the resident immune cells of the central nervous system, and they monitor and clear pathogens. The progression of AD is often accompanied by the inflammatory activation and metabolic reprogramming of microglia [101]. Pan et al. [102] found that histone lactylation (H4K12la) increased in microglia adjacent to plaque in the brain of 5XFAD model mice and AD patients. They found that glycolysis in microglia is active when AD occurs, and a large amount of lactic acid is produced to promote the modification of H4K12la; this modification can improve the transcription level of multiple glycolytic genes in microglia and further enhance glycolysis in microglia, thus forming a glycolysis/H4K12la/PKM2 positive feedback cycle. The activation of the positive feedback loop aggravates microglial dysfunction. When the loop is interrupted, it can improve microglial dysfunction and Aβ pathology.

### 7.5. Histone Lactylation and Gut Microbe

Lactic acid has been proven to mediate histone lactylation, regulate gene transcription in tumor-related immune cells, and help tumor cells achieve immune escape [3]. Current research has shown that lactate modification plays a significant role in inflammation, fibrosis, stem cell differentiation, and tumors [3]. Histone lysine lactate modification, as a non-metabolic function of lactate, is involved in pathological processes such as infection and tumor development. In the normal organism, the intestinal mucosal immune system can sense changes in gut microbes and transmit signals to M cells and Paneth cells to release cathelicidin and other substances, effectively eliminating pathogenic microorganisms. Alternatively, signals can be transmitted to immune cells, dendritic cells, NK cells, or macrophages to release anti-inflammatory cytokines to eliminate harmful substances [103].

Gut microbes interact with the host genome through epigenetic mechanisms, such as long non-coding RNAs (lncRNAs), and participate in pathogenesis [104,105]. In colorectal cancer (CRC) or colorectal adenomas, Xia et al. found new associations between the abundance of tumor-related bacteria and the universal hypermethylation of the promoter of a tumor suppressor gene [106]. Stimulation of human primary monocytes with lipopolysaccharide (LPS), the main cell wall component of Gram-negative bacteria, induces the expression of lncRNAs and then regulates the host immune response [107]. Research reports suggested that LPS can increase lactate levels in mouse models and human cell lines [3,108].

LINC00152 is overexpressed in a variety of tumors, including breast cancer, pancreatic cancer, and CRC [109,110,111]. Elevated expression of LINC00152 is considered a risk factor for invasion and metastasis in tumor patients and is associated with poor prognosis [112]. Studies have shown that Salmonella infection can induce activation of the NF-κB pathway [113,114]. Overexpression of LINC00152 reduces the expression of IL-8 and TNF-α, targets of the NF-κB pathway. On the other hand, Salmonella infection injects its effector factors into host cells through the type III secretion system (T3SS) [115]. Wang et al. found an increase in lactate levels in HCT116 cells stimulated by LPS and a significant increase in LINC00152 expression in HCT116 cells treated with lactate [116]. Further research found that LPS reduced the binding efficiency of inhibitory factor anti-YY1 to LINC00152 by introducing histone lactylation on its promoter, thereby upregulating the expression of LINC00152 [117]. These findings suggest that histone lysine lactylation may be a new mechanism for LPS-induced gene expression changes.

To provide a clearer overview of these diverse functions, a summary of the major roles and mechanisms of histone lactylation in different biological contexts is presented in Table 2.

## 8. Therapeutic Avenues of Targeting Lactylation in Combination Regimens

Emerging evidence demonstrates that modulation of histone lactylation can be effectively integrated into existing therapeutic frameworks, including three main combinatorial strategies: overcoming immunotherapy resistance by targeting lactate-driven immune evasion, reversing chemoresistance via lactylation inhibition, and harnessing synergies between glycolysis or lactate metabolism inhibitors and conventional or epigenetic therapies.

A growing body of evidence indicates that histone lactylation is a major driver of immunotherapy resistance across multiple cancer types. In pancreatic cancer, lactate-induced H3K18la upregulates CXCL1 expression, leading to neutrophil infiltration and establishing an immunosuppressive microenvironment. Combinatorial treatment with bromosporine, a PCAF inhibitor that blocks lactylation, together with an anti-PD-1 antibody, produced synergistic antitumor effects in both subcutaneous and orthotopic pancreatic cancer models [118]. In hepatocellular carcinoma, lactate-driven H3K18la transcriptionally upregulates KIF20A, which stabilizes the c-Myc/PD-L1 axis. Combining glycolysis inhibition (which reduces lactate production) with anti-PD-1 therapy reversed immunosuppression and synergistically suppressed tumor growth [119]. The broader roles of lactylation in reshaping the tumor immunosuppressive microenvironment have been comprehensively reviewed [120]. Together, these studies establish a strong mechanistic link between lactylation-driven immune evasion and immunotherapy resistance, supporting the rationale for combining lactylation modulating agents with immune checkpoint inhibitors [121].

Beyond immunotherapy, targeting lactylation has also been shown to overcome chemoresistance. In docetaxel-resistant prostate cancer, H3K18la directly activates FOXM1 transcription through promoter enrichment. The phytochemical combination of icariin–curcumol (Ica-Cur) restored docetaxel sensitivity by suppressing H3K18la and FOXM1 expression, demonstrating that a relatively safe phytochemical agent can be used to target the lactylation pathway without the toxicity concerns of more conventional small-molecule inhibitors [122]. In clear cell renal cell carcinoma, VHL inactivation triggers a pathogenic positive feedback loop involving H3K18la and PDGFRβ signaling, suggesting that combined inhibition of both pathways can be more effective than targeting either alone [59].

A third promising avenue involves combining lactylation targeting with existing metabolic or epigenetic inhibitors. Notably, the neddylation inhibitor MLN4924 (pevonedistat) has been shown to paradoxically enhance H3K18la through LDH activation yet still suppress breast cancer metastasis via ITGB4 downregulation. This counter-intuitive finding indicates that the relationship between lactylation and therapeutic outcome can be context-dependent, and that pharmacologically modulating lactylation might require careful calibration rather than simply aiming for global inhibition [123]. Meanwhile, broader therapeutic strategies targeting the lactate–lactylation axis without directly inhibiting lactylation itself have also shown translational potential. The lactate/GPR81 signaling axis has been identified as a metabolic checkpoint that suppresses antitumor immunity, with dual blockade of the lactate/GPR81 and PD-1/PD-L1 pathways enhancing the antitumor effects of metformin [121]. In pancreatic cancer, disrupting the lactate-mediated metabolic crosstalk between tumor sub-populations using lactate interceptor strategies has demonstrated the ability to overcome lactate-associated resistance to chemo-immunotherapy. In hepatocellular carcinoma, targeting lactylation events on the mitochondrial protein LRPPRC has been shown to disrupt metabolic–immune crosstalk and restore antitumor immunity, further underscoring the broad applicability of the lactate–lactylation axis as a therapeutic node [124].

Collectively, these preclinical studies provide a strong rationale for integrating lactylation-targeting strategies into combination regimens. It is important to note that translating these promising findings will require addressing several key challenges. The development of selective lactylation inhibitors with minimal off-target effects remains an unmet need, as most existing modulators (glycolysis inhibitors, HDAC inhibitors, and KAT inhibitors) lack the specificity required to distinguish lactylation from other acyl modifications. Additionally, the identification of predictive biomarkers, including lactylation site-specific signatures such as H3K18la levels or BLM K24la status, will be essential for patient stratification in future clinical trials [125]. Finally, rigorous evaluation in well-designed preclinical models, along with careful assessment of potential toxicity associated with long-term lactylation modulation, will be necessary before clinical translation can be considered.

## 9. Conclusions

As a novel post-translational modification, it is particularly important to study the role and regulatory mechanisms of protein lactylation in physiological and pathological processes. Although increasing research suggests that lactylation may become a potential therapeutic target in metabolic disorders, inflammation, tumors, and neurological and psychiatric disorders, there are still several key issues that remain unresolved. Most existing work relies on global changes in lactate levels or overexpression of writing and erasing enzymes, which makes it difficult to attribute functionality to a single lactate modification. Tools that fuse lactyltransferase or deacetylase with dCas9, or nanobodies that can block lactate residue recognition, may help to decipher the causal effects of specific sites. Future research should focus on developing site-specific editing strategies to precisely manipulate specific acetylation sites and analyze their causal biological functions. In addition, it is necessary to establish more sensitive and quantitative analysis methods. Many studies are still using pan-antibodies to report relative changes in lactate through Western blot, which cannot provide stoichiometric information. Establishing a stable isotope internal standard for targeted mass spectrometry can allow for accurately quantifying the occupancy rate of lactylation under different conditions in order to accurately detect the abundance and dynamic changes of lactylation modifications in vivo.

Another important direction is the systematic identification and functional validation of true lactylation “readers,” “writers,” and “erasers,” which are currently not fully understood as regulatory factors. Although writers and erasers have been identified, the readers that specifically bind lactylated histones and convert the modification into transcriptional outcomes remain largely unknown. Systematic pull-down screens using synthetic lactylated peptides combined with quantitative proteomics could fill this gap.

In addition, establishing tissue-specific and cell type-specific animal models is crucial for understanding the context-dependent function of acetylation under different physiological and pathological conditions. Global knockouts of enzymes involved in lactylation often cause severe developmental defects or lethality, obscuring tissue-specific functions. Conditional knockout mice targeting p300, HDACs, or GLO1 in defined cell types (for example, myeloid cells, hepatocytes, or neurons) would provide clearer answers. Knockin mice carrying point mutations that eliminate individual lactylation sites, such as H3K18R, would offer definitive evidence for the physiological relevance of specific marks.

Finally, rigorous in vivo causal studies are needed to determine whether lactylation is a driving factor for disease progression or merely a concomitant phenomenon reflecting potential metabolic changes. Current work is largely correlative. Acute manipulation approaches, such as viral delivery of constitutively active or dominant-negative versions of p300 or HDACs, or in vivo CRISPR activation or interference targeting specific genomic loci, could determine directionality.

These research advances will help promote the gradual transition of lactylation research from phenomenon description to mechanism analysis and clinical translation, and ultimately achieve rational targeting of lactylation in cancer, inflammation, neurodegenerative diseases, and metabolic diseases.

## Figures and Tables

**Table 1:** Major histone lactylation sites reported to date.

Histone	Known Lactylation Sites	Key References
H3	K9, K14, K18, K23, K56, K79	[3,63,64,65]
H4	K5, K8, K12, K16, K79, K91	[3,66]
H2A	Multiple sites (detailed residues under investigation)	[3]
H2B	K16, K58	[67]
Linker H1	Multiple sites (H1Kla)	[68]

**Table 2:** Summary of histone lactylation functions in physiological and pathological processes.

Process/Condition	Lactylation Site	Functional Role/Mechanism	Key Targets/Molecules	References
Cell fate determination	H3K18la, H3K23la, pan-Kla	Glis1-induced metabolic remodeling enhances glycolysis and lactate production, promoting H3K18la and pluripotent gene expression; lactate activates germline and cleavage embryo genes; hypoxia reduces lactylation and impairs preimplantation development; elevated H3K18la in receptive endometrium facilitates embryo implantation	Pluripotent genes (*Oct4*, *Sox2*); germline/embryo genes; endometrial redox- and apoptosis-related genes	[82,84,85,86]
Inflammation and macrophage polarization	Pan-Kla, H3K18la	Late-stage M1 macrophages produce lactate, driving H3K18la and *Arg1* expression to promote M2-like repair phenotype; BCAP-PI3K-AKT axis links glycolysis to lactylation; in pulmonary fibrosis, lactate induces alveolar macrophage lactylation and *Arg1*; in sepsis, HMGB1 lactylation disrupts endothelial barrier	*Arg1*, tissue repair genes; HMGB1; endothelial adhesion/tight junction proteins	[3,8,70,71,94]
Tumorigenesis	H3K18la, H3K9la, H3K56la, pan-Kla	In ocular melanoma, H3K18la drives YTHDF2 expression, degrading PER1 and TP53; in liver cancer stem cells, elevated pan-Kla, H3K9la, and H3K56la correlate with malignancy; in NSCLC, histone lactylation regulates glycolytic/TCA cycle enzymes; in ccRCC, VHL inactivation triggers a positive feedback loop between histone lactylation and PDGFRβ signaling	YTHDF2, PER1, TP53; CD133, BCL2, LDHA; HK-1, PKM, SDHA, IDH3G; PDGFRβ	[59,97,99,100]
Neurodegeneration	H4K12la, histone H1	Social defeat stress upregulates histone H1 lactylation, associated with reduced social behavior; in Alzheimer’s disease, microglial glycolysis generates lactate, promoting H4K12la, which upregulates PKM2 and further enhances glycolysis (positive feedback loop), exacerbating Aβ pathology	Histone H1; PKM2; glycolytic genes	[68,102]
Gut microbe interaction	Histone lactylation (site not specified)	LPS (from Gram-negative bacteria) upregulates lactate levels and induces histone lactylation on the *LINC00152* promoter, reducing YY1 binding and upregulating *LINC00152*, which promotes CRC cell invasion and migration	LINC00152; YY1; NF-κB pathway targets (IL-8, TNF-α)	[116,117]

## Data Availability

No new data were created or analyzed in this study.

## Abbreviations

The following abbreviations are used in this manuscript:

AD	Alzheimer’s disease
AMPK	AMP-activated protein kinase
ANLS	Astrocyte–neuron lactate shuttle
Arg1	Arginase 1
ATM	Adipose tissue macrophage
ATP	Adenosine triphosphate
BCAP	B-cell adapter for PI3K
BCL2	B-cell lymphoma 2
cAMP	Cyclic adenosine monophosphate
CBP	CREB-binding protein
ccRCC	Clear cell renal cell carcinoma
CoA	Coenzyme A
CRC	Colorectal cancer
FOXO1	Forkhead box protein O1
GLO1	Glyoxalase I
GLO2	Glyoxalase II
Glis1	GLIS family zinc finger 1
GPCR	G-protein-coupled receptor
GPR81	G-protein-coupled receptor 81
GPR132	G-protein-coupled receptor 132
GSK3β	Glycogen synthase kinase 3 beta
HAT	Histone acetyltransferase
HCAR	Hydroxy-carboxylic acid receptor
HCC	Hepatocellular carcinoma
HDAC	Histone deacetylase
HFD	High-fat diet
HIF	Hypoxia-inducible factor
HIF-1α	Hypoxia-inducible factor 1-alpha
HMGB1	High mobility group box 1
IDH3G	Isocitrate dehydrogenase 3 gamma
IFNγ	Interferon-gamma
IL-6	Interleukin-6
IL-8	Interleukin-8
iPSC	Induced pluripotent stem cell
Kla	Lysine lactylation
LCSC	Liver cancer stem cell
LDH	Lactate dehydrogenase
LDHA	Lactate dehydrogenase A
LGSH	Lactoylglutathione
LINC00152	Long intergenic non-protein coding RNA 152
lncRNA	Long non-coding RNA
LPS	Lipopolysaccharide
MCT	Monocarboxylate transporter
MMP-2	Matrix metallopeptidase-2
NAD^+^	Nicotinamide adenine dinucleotide
NADH	Nicotinamide adenine dinucleotide (reduced)
NF-κB	Nuclear factor kappa-light-chain-enhancer of activated B cells
OXPHOS	Oxidative phosphorylation
P300	Histone acetyltransferase p300 (EP300)
PDC	Pyruvate dehydrogenase complex
PDGFRβ	Platelet-derived growth factor receptor beta
PDH	Pyruvate dehydrogenase
PER1	Period circadian regulator 1
PI3K	Phosphoinositide 3-kinase
PKA	Protein kinase A
PKM	Pyruvate kinase M
PKM2	Pyruvate kinase M2
PRR	Pattern recognition receptor
PTM	Post-translational modification
SDHA	Succinate dehydrogenase complex flavoprotein subunit A
SirT	Sirtuin
STAT3	Signal transducer and activator of transcription 3
T3SS	Type III secretion system
TCA	Tricarboxylic acid cycle
TGFβ2	Transforming growth factor beta 2
TLR	Toll-like receptor
TNF-α	Tumor necrosis factor alpha
TP53	Tumor protein p53
VEGFA	Vascular endothelial growth factor A
VHL	Von Hippel–Lindau
YTHDF2	YTH N6-methyladenosine RNA binding protein 2
YY1	Yin Yang 1

## References

[1] Xie Y., Hu H., Liu M., Zhou T., Cheng X., Huang W., Cao L. (2022). The role and mechanism of histone lactylation in health and diseases. Front. Genet..

[2] Ye L., Jiang Y., Zhang M. (2022). Crosstalk between glucose metabolism, lactate production and immune response modulation. Cytokine Growth Factor Rev..

[3] Zhang D., Tang Z., Huang H., Zhou G., Cui C., Weng Y., Liu W., Kim S., Lee S., Perez-Neut M. (2019). Metabolic regulation of gene expression by histone lactylation. Nature.

[4] Peng X., Du J. (2025). Histone and non-histone lactylation: Molecular mechanisms, biological functions, diseases, and therapeutic targets. Mol. Biomed..

[5] Shi P., Ma Y., Zhang S. (2025). Non-histone lactylation: Unveiling its functional significance. Front. Cell Dev. Biol..

[6] Sheng X., Lin H., Cole P.A., Zhao Y. (2026). Biochemistry and regulation of histone lysine L-lactylation. Nat. Rev. Mol. Cell Biol..

[7] Colegio O.R., Chu N.Q., Szabo A.L., Chu T., Rhebergen A.M., Jairam V., Cyrus N., Brokowski C.E., Eisenbarth S.C., Phillips G.M. (2014). Functional polarization of tumour-associated macrophages by tumour-derived lactic acid. Nature.

[8] Irizarry-Caro R.A., McDaniel M.M., Overcast G.R., Jain V.G., Troutman T.D., Pasare C. (2020). TLR signaling adapter BCAP regulates inflammatory to reparatory macrophage transition by promoting histone lactylation. Proc. Natl. Acad. Sci. USA.

[9] Ouyang Q., Hu Q., Wang C., He Y., Zeng R., Li Y., Su C., Lu G., Zhu X., Xiao L. (2026). Protein Lactylation in Cancer: Mechanisms and Therapeutic Targets. MedComm.

[10] Li J., Ma P., Liu Z., Xie J. (2025). L- and D-Lactate: Unveiling their hidden functions in disease and health. Cell Commun. Signal..

[11] Pohanka M. (2020). D-Lactic Acid as a Metabolite: Toxicology, Diagnosis, and Detection. Biomed. Res. Int..

[12] Brooks G.A. (2009). Cell-cell and intracellular lactate shuttles. J. Physiol..

[13] Shi W., Cassmann T.J., Bhagwate A.V., Hitosugi T., Ip W.K.E. (2024). Lactic acid induces transcriptional repression of macrophage inflammatory response via histone acetylation. Cell Rep..

[14] Vander Heiden M.G., Cantley L.C., Thompson C.B. (2009). Understanding the Warburg effect: The metabolic requirements of cell proliferation. Science.

[15] Prochownik E.V., Wang H. (2021). The Metabolic Fates of Pyruvate in Normal and Neoplastic Cells. Cells.

[16] Vaupel P., Schmidberger H., Mayer A. (2019). The Warburg effect: Essential part of metabolic reprogramming and central contributor to cancer progression. Int. J. Radiat. Biol..

[17] Urbano A.M. (2021). Otto Warburg: The journey towards the seminal discovery of tumor cell bioenergetic reprogramming. Biochim. Biophys. Acta Mol. Basis Dis..

[18] Watts J.A., Kline J.A. (2003). Bench to bedside: The role of mitochondrial medicine in the pathogenesis and treatment of cellular injury. Acad. Emerg. Med..

[19] Fisher-Wellman K.H., Neufer P.D. (2012). Linking mitochondrial bioenergetics to insulin resistance via redox biology. Trends Endocrinol. Metab..

[20] Adeva-Andany M., Lopez-Ojen M., Funcasta-Calderon R., Ameneiros-Rodriguez E., Donapetry-Garcia C., Vila-Altesor M., Rodriguez-Seijas J. (2014). Comprehensive review on lactate metabolism in human health. Mitochondrion.

[21] Perriello G., Jorde R., Nurjhan N., Stumvoll M., Dailey G., Jenssen T., Bier D.M., Gerich J.E. (1995). Estimation of glucose-alanine-lactate-glutamine cycles in postabsorptive humans: Role of skeletal muscle. Am. J. Physiol..

[22] Kreisberg R.A. (1980). Lactate homeostasis and lactic acidosis. Ann. Intern. Med..

[23] Perez-Escuredo J., Van Hee V.F., Sboarina M., Falces J., Payen V.L., Pellerin L., Sonveaux P. (2016). Monocarboxylate transporters in the brain and in cancer. Biochim. Biophys. Acta.

[24] Sun S., Li H., Chen J., Qian Q. (2017). Lactic Acid: No Longer an Inert and End-Product of Glycolysis. Physiology.

[25] Halestrap A.P., Meredith D. (2004). The SLC16 gene family-from monocarboxylate transporters (MCTs) to aromatic amino acid transporters and beyond. Pflug. Arch..

[26] Dhup S., Dadhich R.K., Porporato P.E., Sonveaux P. (2012). Multiple biological activities of lactic acid in cancer: Influences on tumor growth, angiogenesis and metastasis. Curr. Pharm. Des..

[27] Ullah M.S., Davies A.J., Halestrap A.P. (2006). The plasma membrane lactate transporter MCT4, but not MCT1, is up-regulated by hypoxia through a HIF-1alpha-dependent mechanism. J. Biol. Chem..

[28] Sonveaux P., Vegran F., Schroeder T., Wergin M.C., Verrax J., Rabbani Z.N., De Saedeleer C.J., Kennedy K.M., Diepart C., Jordan B.F. (2008). Targeting lactate-fueled respiration selectively kills hypoxic tumor cells in mice. J. Clin. Investig..

[29] Baltazar F., Pinheiro C., Morais-Santos F., Azevedo-Silva J., Queiros O., Preto A., Casal M. (2014). Monocarboxylate transporters as targets and mediators in cancer therapy response. Histol. Histopathol..

[30] Lee D., Wu A., Yao L., Satish S., Mei L., Xiong W.C. (2026). Neuronal MCT2 promotes angiogenesis via lactate in the developing mouse neocortex. Cell Death Differ..

[31] Philp N.J., Yoon H., Lombardi L. (2001). Mouse MCT3 gene is expressed preferentially in retinal pigment and choroid plexus epithelia. Am. J. Physiol. Cell Physiol..

[32] Yoon H., Fanelli A., Grollman E.F., Philp N.J. (1997). Identification of a unique monocarboxylate transporter (MCT3) in retinal pigment epithelium. Biochem. Biophys. Res. Commun..

[33] Adijanto J., Philp N.J. (2012). The SLC16A family of monocarboxylate transporters (MCTs)--physiology and function in cellular metabolism, pH homeostasis, and fluid transport. Curr. Top. Membr..

[34] Felmlee M.A., Jones R.S., Rodriguez-Cruz V., Follman K.E., Morris M.E. (2020). Monocarboxylate Transporters (SLC16): Function, Regulation, and Role in Health and Disease. Pharmacol. Rev..

[35] Ahmed K., Tunaru S., Offermanns S. (2009). GPR109A, GPR109B and GPR81, a family of hydroxy-carboxylic acid receptors. Trends Pharmacol. Sci..

[36] Offermanns S., Colletti S.L., Lovenberg T.W., Semple G., Wise A., IJzerman A.P. (2011). International Union of Basic and Clinical Pharmacology. LXXXII: Nomenclature and Classification of Hydroxy-carboxylic Acid Receptors (GPR81, GPR109A, and GPR109B). Pharmacol. Rev..

[37] Liu C., Wu J., Zhu J., Kuei C., Yu J., Shelton J., Sutton S.W., Li X., Yun S.J., Mirzadegan T. (2009). Lactate inhibits lipolysis in fat cells through activation of an orphan G-protein-coupled receptor, GPR81. J. Biol. Chem..

[38] Cai T.Q., Ren N., Jin L., Cheng K., Kash S., Chen R., Wright S.D., Taggart A.K., Waters M.G. (2008). Role of GPR81 in lactate-mediated reduction of adipose lipolysis. Biochem. Biophys. Res. Commun..

[39] Morland C., Andersson K.A., Haugen O.P., Hadzic A., Kleppa L., Gille A., Rinholm J.E., Palibrk V., Diget E.H., Kennedy L.H. (2017). Exercise induces cerebral VEGF and angiogenesis via the lactate receptor HCAR1. Nat. Commun..

[40] Feingold K.R., Moser A., Shigenaga J.K., Grunfeld C. (2011). Inflammation inhibits GPR81 expression in adipose tissue. Inflamm. Res..

[41] Rooney K., Trayhurn P. (2011). Lactate and the GPR81 receptor in metabolic regulation: Implications for adipose tissue function and fatty acid utilisation by muscle during exercise. Br. J. Nutr..

[42] Rabinowitz J.D., Enerback S. (2020). Lactate: The ugly duckling of energy metabolism. Nat. Metab..

[43] Brooks G.A. (2018). The Science and Translation of Lactate Shuttle Theory. Cell Metab..

[44] Magistretti P.J., Pellerin L. (1996). The contribution of astrocytes to the 18F-2-deoxyglucose signal in PET activation studies. Mol. Psychiatry.

[45] Schurr A., Gozal E. (2011). Aerobic production and utilization of lactate satisfy increased energy demands upon neuronal activation in hippocampal slices and provide neuroprotection against oxidative stress. Front. Pharmacol..

[46] Magistretti P.J., Allaman I. (2018). Lactate in the brain: From metabolic end-product to signalling molecule. Nat. Rev. Neurosci..

[47] van Hall G., Stromstad M., Rasmussen P., Jans O., Zaar M., Gam C., Quistorff B., Secher N.H., Nielsen H.B. (2009). Blood lactate is an important energy source for the human brain. J. Cereb. Blood Flow. Metab..

[48] Overgaard M., Rasmussen P., Bohm A.M., Seifert T., Brassard P., Zaar M., Homann P., Evans K.A., Nielsen H.B., Secher N.H. (2012). Hypoxia and exercise provoke both lactate release and lactate oxidation by the human brain. FASEB J..

[49] Pellerin L., Magistretti P.J. (1994). Glutamate uptake into astrocytes stimulates aerobic glycolysis: A mechanism coupling neuronal activity to glucose utilization. Proc. Natl. Acad. Sci. USA.

[50] Carrard A., Elsayed M., Margineanu M., Boury-Jamot B., Fragniere L., Meylan E.M., Petit J.M., Fiumelli H., Magistretti P.J., Martin J.L. (2018). Peripheral administration of lactate produces antidepressant-like effects. Mol. Psychiatry.

[51] Takahashi H., Alves C.R.R., Stanford K.I., Middelbeek R.J.W., Nigro P., Ryan R.E., Xue R., Sakaguchi M., Lynes M.D., So K. (2019). TGF-beta2 is an exercise-induced adipokine that regulates glucose and fatty acid metabolism. Nat. Metab..

[52] Cai H., Wang X., Zhang Z., Chen J., Wang F., Wang L., Liu J. (2022). Moderate l-lactate administration suppresses adipose tissue macrophage M1 polarization to alleviate obesity-associated insulin resistance. J. Biol. Chem..

[53] Ge X., Wang S., Li Z., Yu J., Liu B., Wang R., Bu S., Wan N., Wang Y., Dai C. (2025). Lactate-Activated GPR132-Src Signal Induces Macrophage Senescence and Aggravates Atherosclerosis Under Diabetes. Adv. Sci..

[54] Zhang Y., Jia P., Wang K., Zhang Y., Lv Y., Fan P., Yang L., Zhang S., Wang T., Zhao J. (2023). Lactate modulates microglial inflammatory responses after oxygen-glucose deprivation through HIF-1alpha-mediated inhibition of NF-kappaB. Brain Res. Bull..

[55] Han S., Bao X., Zou Y., Wang L., Li Y., Yang L., Liao A., Zhang X., Jiang X., Liang D. (2023). d-lactate modulates M2 tumor-associated macrophages and remodels immunosuppressive tumor microenvironment for hepatocellular carcinoma. Sci. Adv..

[56] Gaffney D.O., Jennings E.Q., Anderson C.C., Marentette J.O., Shi T., Schou Oxvig A.M., Streeter M.D., Johannsen M., Spiegel D.A., Chapman E. (2020). Non-enzymatic Lysine Lactoylation of Glycolytic Enzymes. Cell Chem. Biol..

[57] Varner E.L., Trefely S., Bartee D., von Krusenstiern E., Izzo L., Bekeova C., O’Connor R.S., Seifert E.L., Wellen K.E., Meier J.L. (2020). Quantification of lactoyl-CoA (lactyl-CoA) by liquid chromatography mass spectrometry in mammalian cells and tissues. Open Biol..

[58] Huang W., Wang Z., Lei Q.Y. (2014). Acetylation control of metabolic enzymes in cancer: An updated version. Acta Biochim. Biophys. Sin..

[59] Yang J., Luo L., Zhao C., Li X., Wang Z., Zeng Z., Yang X., Zheng X., Jie H., Kang L. (2022). A Positive Feedback Loop between Inactive VHL-Triggered Histone Lactylation and PDGFRbeta Signaling Drives Clear Cell Renal Cell Carcinoma Progression. Int. J. Biol. Sci..

[60] Li J., Li Z., Zhang X., Zhang H., Tan M., Tang Y., Guo S., Ye T., Wang J., Li J. (2026). Histone lactylation bridges metabolic reprogramming with chromatin-immune crosstalk in triple-negative breast cancer. Cancer Lett..

[61] Galle E., Wong C.W., Ghosh A., Desgeorges T., Melrose K., Hinte L.C., Castellano-Castillo D., Engl M., de Sousa J.A., Ruiz-Ojeda F.J. (2022). H3K18 lactylation marks tissue-specific active enhancers. Genome Biol..

[62] Ma Y., Zhang Z., Cao X., Guo D., Huang S., Xie L., Wu M., Li J., Li C., Chu Y. (2025). Semaphorin 6A phase separation sustains a histone lactylation-dependent lactate buildup in pathological angiogenesis. Proc. Natl. Acad. Sci. USA.

[63] Huang Y., Wang C., Zhou T., Xie F., Liu Z., Xu H., Liu M., Wang S., Li L., Chi Q. (2024). Lumican promotes calcific aortic valve disease through H3 histone lactylation. Eur. Heart J..

[64] Zhao P., Qiao C., Wang J., Zhou Y., Zhang C. (2024). Histone lactylation facilitates hepatocellular carcinoma progression by upregulating endothelial cell-specific molecule 1 expression. Mol. Carcinog..

[65] Xu H., Li L., Wang S., Wang Z., Qu L., Wang C., Xu K. (2023). Royal jelly acid suppresses hepatocellular carcinoma tumorigenicity by inhibiting H3 histone lactylation at H3K9la and H3K14la sites. Phytomedicine.

[66] Liu J., Zhao L., Yan M., Jin S., Shang L., Wang J., Wang Q., Zhao S., Shen Z., Liu T. (2025). H4K79 and H4K91 histone lactylation, newly identified lactylation sites enriched in breast cancer. J. Exp. Clin. Cancer Res..

[67] Zhu W., Zeng S., Zhu S., Zhang Z., Zhao R., Qiu Q., Luo Z., Qin Y., Chen W., Li B. (2025). Histone H2B lysine lactylation modulates the NF-kappaB response via KPNA2 during CSFV infection. Int. J. Biol. Macromol..

[68] Hagihara H., Shoji H., Otabi H., Toyoda A., Katoh K., Namihira M., Miyakawa T. (2021). Protein lactylation induced by neural excitation. Cell Rep..

[69] Tsusaka T., Najar M.A., Schwarz B., Bohrnsen E., Oses-Prieto J.A., Neudorf H., Lee C., Little J.P., Burlingame A.L., Bosio C.M. (2025). Reversible histone deacetylase activity catalyzes lysine acylation. Nat. Chem. Biol..

[70] Cui H., Xie N., Banerjee S., Ge J., Jiang D., Dey T., Matthews Q.L., Liu R.M., Liu G. (2021). Lung Myofibroblasts Promote Macrophage Profibrotic Activity through Lactate-induced Histone Lactylation. Am. J. Respir. Cell Mol. Biol..

[71] Yang K., Fan M., Wang X., Xu J., Wang Y., Tu F., Gill P.S., Ha T., Liu L., Williams D.L. (2022). Lactate promotes macrophage HMGB1 lactylation, acetylation, and exosomal release in polymicrobial sepsis. Cell Death Differ..

[72] Su X., Wellen K.E., Rabinowitz J.D. (2016). Metabolic control of methylation and acetylation. Curr. Opin. Chem. Biol..

[73] Moreno-Yruela C., Zhang D., Wei W., Baek M., Liu W., Gao J., Dankova D., Nielsen A.L., Bolding J.E., Yang L. (2022). Class I histone deacetylases (HDAC1-3) are histone lysine delactylases. Sci. Adv..

[74] Zhang D., Gao J., Zhu Z., Mao Q., Xu Z., Singh P.K., Rimayi C.C., Moreno-Yruela C., Xu S., Li G. (2025). Lysine L-lactylation is the dominant lactylation isomer induced by glycolysis. Nat. Chem. Biol..

[75] Zhang C., Zhou T., Li C., Wang D., Tao J., Zhu X., Lu J., Ni J., Yao Y.F. (2025). Deciphering novel enzymatic and non-enzymatic lysine lactylation in Salmonella. Emerg. Microbes Infect..

[76] Jennings E.Q., Ray J.D., Zerio C.J., Trujillo M.N., McDonald D.M., Chapman E., Spiegel D.A., Galligan J.J. (2021). Sirtuin 2 Regulates Protein LactoylLys Modifications. Chembiochem.

[77] Xu H., Wu M., Ma X., Huang W., Xu Y. (2021). Function and Mechanism of Novel Histone Posttranslational Modifications in Health and Disease. Biomed. Res. Int..

[78] Zhou T., Cheng X., He Y., Xie Y., Xu F., Xu Y., Huang W. (2022). Function and mechanism of histone beta-hydroxybutyrylation in health and disease. Front. Immunol..

[79] Ghosh-Choudhary S., Liu J., Finkel T. (2020). Metabolic Regulation of Cell Fate and Function. Trends Cell Biol..

[80] Han J.K., Shin Y., Kim H.S. (2022). Direct Conversion of Cell Fate and Induced Endothelial Cells. Circ. J..

[81] Liu Y., Cui D.X., Pan Y., Yu S.H., Zheng L.W., Wan M. (2022). Metabolic-epigenetic nexus in regulation of stem cell fate. World J. Stem Cells.

[82] Li L., Chen K., Wang T., Wu Y., Xing G., Chen M., Hao Z., Zhang C., Zhang J., Ma B. (2020). Glis1 facilitates induction of pluripotency via an epigenome-metabolome-epigenome signalling cascade. Nat. Metab..

[83] Maekawa M., Yamaguchi K., Nakamura T., Shibukawa R., Kodanaka I., Ichisaka T., Kawamura Y., Mochizuki H., Goshima N., Yamanaka S. (2011). Direct reprogramming of somatic cells is promoted by maternal transcription factor Glis1. Nature.

[84] Tian Q., Zhou L.Q. (2022). Lactate Activates Germline and Cleavage Embryo Genes in Mouse Embryonic Stem Cells. Cells.

[85] Yang W., Wang P., Cao P., Wang S., Yang Y., Su H., Nashun B. (2021). Hypoxic in vitro culture reduces histone lactylation and impairs pre-implantation embryonic development in mice. Epigenet. Chromatin.

[86] Yang Q., Liu J., Wang Y., Zhao W., Wang W., Cui J., Yang J., Yue Y., Zhang S., Chu M. (2022). A proteomic atlas of ligand-receptor interactions at the ovine maternal-fetal interface reveals the role of histone lactylation in uterine remodeling. J. Biol. Chem..

[87] Medzhitov R. (2008). Origin and physiological roles of inflammation. Nature.

[88] Ding H., Wang J.J., Zhang X.Y., Yin L., Feng T. (2021). Lycium barbarum Polysaccharide Antagonizes LPS-Induced Inflammation by Altering the Glycolysis and Differentiation of Macrophages by Triggering the Degradation of PKM2. Biol. Pharm. Bull..

[89] Fitzgerald K.A., Kagan J.C. (2020). Toll-like Receptors and the Control of Immunity. Cell.

[90] Martin M., Rehani K., Jope R.S., Michalek S.M. (2005). Toll-like receptor-mediated cytokine production is differentially regulated by glycogen synthase kinase 3. Nat. Immunol..

[91] Fan W., Morinaga H., Kim J.J., Bae E., Spann N.J., Heinz S., Glass C.K., Olefsky J.M. (2010). FoxO1 regulates Tlr4 inflammatory pathway signalling in macrophages. EMBO J..

[92] Saline M., Badertscher L., Wolter M., Lau R., Gunnarsson A., Jacso T., Norris T., Ottmann C., Snijder A. (2019). AMPK and AKT protein kinases hierarchically phosphorylate the N-terminus of the FOXO1 transcription factor, modulating interactions with 14-3-3 proteins. J. Biol. Chem..

[93] Cross D.A., Alessi D.R., Cohen P., Andjelkovich M., Hemmings B.A. (1995). Inhibition of glycogen synthase kinase-3 by insulin mediated by protein kinase B. Nature.

[94] Chu X., Di C., Chang P., Li L., Feng Z., Xiao S., Yan X., Xu X., Li H., Qi R. (2021). Lactylated Histone H3K18 as a Potential Biomarker for the Diagnosis and Predicting the Severity of Septic Shock. Front. Immunol..

[95] Dichtl S., Lindenthal L., Zeitler L., Behnke K., Schlosser D., Strobl B., Scheller J., El Kasmi K.C., Murray P.J. (2021). Lactate and IL6 define separable paths of inflammatory metabolic adaptation. Sci. Adv..

[96] Hanahan D., Weinberg R.A. (2011). Hallmarks of cancer: The next generation. Cell.

[97] Yu J., Chai P., Xie M., Ge S., Ruan J., Fan X., Jia R. (2021). Histone lactylation drives oncogenesis by facilitating m(6)A reader protein YTHDF2 expression in ocular melanoma. Genome Biol..

[98] Fan S.T., Yang Z.F., Ho D.W., Ng M.N., Yu W.C., Wong J. (2011). Prediction of posthepatectomy recurrence of hepatocellular carcinoma by circulating cancer stem cells: A prospective study. Ann. Surg..

[99] Pan L., Feng F., Wu J., Fan S., Han J., Wang S., Yang L., Liu W., Wang C., Xu K. (2022). Demethylzeylasteral targets lactate by inhibiting histone lactylation to suppress the tumorigenicity of liver cancer stem cells. Pharmacol. Res..

[100] Jiang J., Huang D., Jiang Y., Hou J., Tian M., Li J., Sun L., Zhang Y., Zhang T., Li Z. (2021). Lactate Modulates Cellular Metabolism Through Histone Lactylation-Mediated Gene Expression in Non-Small Cell Lung Cancer. Front. Oncol..

[101] Hu Y., Mai W., Chen L., Cao K., Zhang B., Zhang Z., Liu Y., Lou H., Duan S., Gao Z. (2020). mTOR-mediated metabolic reprogramming shapes distinct microglia functions in response to lipopolysaccharide and ATP. Glia.

[102] Pan R.Y., He L., Zhang J., Liu X., Liao Y., Gao J., Liao Y., Yan Y., Li Q., Zhou X. (2022). Positive feedback regulation of microglial glucose metabolism by histone H4 lysine 12 lactylation in Alzheimer’s disease. Cell Metab..

[103] Li M.X., Li M.Y., Lei J.X., Wu Y.Z., Li Z.H., Chen L.M., Zhou C.L., Su J.Y., Huang G.X., Huang X.Q. (2022). Huangqin decoction ameliorates DSS-induced ulcerative colitis: Role of gut microbiota and amino acid metabolism, mTOR pathway and intestinal epithelial barrier. Phytomedicine.

[104] Ponziani F.R., Bhoori S., Castelli C., Putignani L., Rivoltini L., Del Chierico F., Sanguinetti M., Morelli D., Paroni Sterbini F., Petito V. (2019). Hepatocellular Carcinoma Is Associated With Gut Microbiota Profile and Inflammation in Nonalcoholic Fatty Liver Disease. Hepatology.

[105] Zhong H., Ren H., Lu Y., Fang C., Hou G., Yang Z., Chen B., Yang F., Zhao Y., Shi Z. (2019). Distinct gut metagenomics and metaproteomics signatures in prediabetics and treatment-naive type 2 diabetics. EBioMedicine.

[106] Xia X., Wu W.K.K., Wong S.H., Liu D., Kwong T.N.Y., Nakatsu G., Yan P.S., Chuang Y.M., Chan M.W., Coker O.O. (2020). Bacteria pathogens drive host colonic epithelial cell promoter hypermethylation of tumor suppressor genes in colorectal cancer. Microbiome.

[107] Ilott N.E., Heward J.A., Roux B., Tsitsiou E., Fenwick P.S., Lenzi L., Goodhead I., Hertz-Fowler C., Heger A., Hall N. (2014). Long non-coding RNAs and enhancer RNAs regulate the lipopolysaccharide-induced inflammatory response in human monocytes. Nat. Commun..

[108] Sommer O., Aug R.L., Schmidt A.J., Heiser P., Schulz E., Vedder H., Clement H.W. (2018). Hydrogen Sulfide Affects Radical Formation in the Hippocampus of LPS Treated Rats and the Effect of Antipsychotics on Hydrogen Sulfide Forming Enzymes in Human Cell Lines. Front. Psychiatry.

[109] Wang X., Yu H., Sun W., Kong J., Zhang L., Tang J., Wang J., Xu E., Lai M., Zhang H. (2018). The long non-coding RNA CYTOR drives colorectal cancer progression by interacting with NCL and Sam68. Mol. Cancer.

[110] Liu Y., Li M., Yu H., Piao H. (2020). lncRNA CYTOR promotes tamoxifen resistance in breast cancer cells via sponging miR-125a-5p. Int. J. Mol. Med..

[111] Zhu H., Shan Y., Ge K., Lu J., Kong W., Jia C. (2020). LncRNA CYTOR promotes pancreatic cancer cell proliferation and migration by sponging miR-205-5p. Pancreatology.

[112] Liang J., Wei X., Liu Z., Cao D., Tang Y., Zou Z., Zhou C., Lu Y. (2018). Long noncoding RNA CYTOR in cancer: A TCGA data review. Clin. Chim. Acta.

[113] Kobelt D., Zhang C., Clayton-Lucey I.A., Glauben R., Voss C., Siegmund B., Stein U. (2020). Pro-inflammatory TNF-alpha and IFN-gamma Promote Tumor Growth and Metastasis via Induction of MACC1. Front. Immunol..

[114] Wang Z., Ao X., Shen Z., Ao L., Wu X., Pu C., Guo W., Xing W., He M., Yuan H. (2021). TNF-alpha augments CXCL10/CXCR3 axis activity to induce Epithelial-Mesenchymal Transition in colon cancer cell. Int. J. Biol. Sci..

[115] Knodler L.A., Elfenbein J.R. (2019). Salmonella enterica. Trends Microbiol..

[116] Wang J., Liu Z., Xu Y., Wang Y., Wang F., Zhang Q., Ni C., Zhen Y., Xu R., Liu Q. (2022). Enterobacterial LPS-inducible LINC00152 is regulated by histone lactylation and promotes cancer cells invasion and migration. Front. Cell Infect. Microbiol..

[117] Chiariotti L., Coretti L., Pero R., Lembo F. (2016). Epigenetic Alterations Induced by Bacterial Lipopolysaccharides. Adv. Exp. Med. Biol..

[118] Zhang P., Ma J., Wan Y., Li C., Liu L., He M., Zhang N., Ma Y., Hu J., Zhao L. (2026). Histone lactylation increases CXCL1 expression for neutrophil infiltration and immune escape in pancreatic cancer. Nat. Commun..

[119] Chen S., Zhao L., Miao T., Han P., Liu J., Hou J., Zhao Q., Wang F., Li J. (2026). Targeting KIF20A blocks lactylation modification to suppress immune escape in hepatocellular carcinoma. iScience.

[120] Yuan Y., Gao Y., Xue J., Zhang H., Liang R., Huang L., Wang Z., Song L., Qin Y., Ai J. (2026). Lactate metabolic reprogramming and lactylation modification: Molecular mechanisms reshaping the tumor immunosuppressive microenvironment. Cell Biosci..

[121] Wang X., Xiong D., Cui S., Duan B., Ding G., Huang Y., Wang Q. (2026). Lactate signaling and immune suppression in tumors: Mechanisms and therapeutic implications. Cell Oncol..

[122] Sheng W., Li Y., Tan T., Yu X., Huang X., Li L., Zhang C., Chen Y., Liu L., Feng M. (2025). Icariin-curcumol inhibits histone H3K18 lactylation and FOXM1 expression to enhance the sensitivity of prostate cancer cells to docetaxel. Cancer Cell Int..

[123] Yu H., Zhou Q., Chen Y., Zhong C., Fu T., Xiong X., Zhu F., Wang L., Sun Y. (2025). Neddylation inhibitor MLN4924 enhances H3K18 lactylation via binding to LDH and downregulates ITGB4 to block metastasis. J. Biol. Chem..

[124] Li N., He Q., Huang Q., Zhu Y. (2026). Targeting LRPPRC lactylation disrupts metabolic-immune crosstalk and restores antitumor immunity in hepatocellular carcinoma. Transl. Cancer Res..

[125] Yang C., Yang R., Zheng B., Jiang H., Wang X., Huang W. (2026). Lactylation as a metabolic-epigenetic switch in cancer: Dual roles in cell death resistance and therapeutic vulnerability. Cell Death Dis..

